# Embryonic exposure to ethanol increases the susceptibility of larval zebrafish to chemically induced seizures

**DOI:** 10.1038/s41598-018-20288-2

**Published:** 2018-01-30

**Authors:** Keling Wang, Xiaopan Chen, Jie Liu, Li-Ping Zou, Wenke Feng, Lu Cai, Xiaoyang Wu, Shao-yu Chen

**Affiliations:** 1 Department of Pharmacology and Toxicology, Alcohol Research Center, University of Louisville Health Science Center, Louisville, KY 40292 USA; 2 Department of Pediatrics, Chinese PLA General Hospital, Beijing, 100853 China; 3 Department of Reproductive Endocrinology, Zhejiang Provincial People’s Hospital, 158 Shangtang Road, Hangzhou, Zhejiang 310014 China; 4 Department of Medicine, University of Louisville, Louisville, KY 40292 USA; 5 Pediatric Research Institute, Department of Pediatrics, University of Louisville, Louisville, KY 40202 USA; 6 Ben May Department for Cancer Research, University of Chicago, Chicago, IL 60637 USA

## Abstract

Prenatal ethanol exposure is known to cause neurodevelopmental disorders. While high prevalence of epilepsy is observed among the children whose mothers abused alcohol during pregnancy, the results from animal studies are conflicting. Here, we investigated whether embryonic exposure to ethanol can increase the susceptibility to pentylenetetrazole (PTZ)-induced seizures in larval zebrafish. Embryos at 3 hours post-fertilization (hpf) were exposed to ethanol at the concentrations ranging from 0.25% to 1% for 21 hours. Control and ethanol-exposed larvae were challenged with PTZ at 7 days post-fertilization (dpf) at the concentrations of 2.5, 5 or 15 mM. The seizure behavior of larvae was recorded and analyzed using EthoVision XT 11. We found that embryonic ethanol exposure increased the percentage of larvae exhibiting typical stage II and III seizure and resulted in a significant reduction in stage I, II and III seizure latency in an ethanol concentration-dependent manner. Embryonic exposure to ethanol also significantly increased the severity of PTZ-induced seizures in larvae, as demonstrated by increased total distance traveled and the duration of mobility. This is the first demonstration that ethanol exposure during early embryonic stage can reduce the threshold for chemically induced seizures and increase the severity of seizure behavior in larval fish.

## Introduction

Fetal Alcohol Spectrum Disorders (FASD) is an umbrella term to describe the disorders that can occur in a child prenatally exposed to alcohol. FASD includes Fetal alcohol syndrome (FAS), Alcohol-Related Neurodevelopment Disorders (ARND) and Alcohol-Related Birth Defects (ARBD) as well as other disorders associated with prenatal alcohol exposure^1,2^. Prenatal ethanol exposure results in brain damage, leading to a variety of neurological disorders and behavioral problems, including epilepsy.

Epilepsy is a prominent manifestation of neurologic dysfunction in many children prenatally exposed to alcohol^3–8^. In a recent prevalence analysis, Bell found that approximately 20% children with FAS have seizure disorders^9^. Among children who were prenatally exposed to alcohol, but without a diagnosis of FAS, the prevalence of seizure disorders range from 14.1% for partial FAS to 18.23% for the alcohol-related neurodevelopmental disorders^9^. It has also been estimated that the prevalence of seizure disorders range from 6% to 21% in individuals with a FAS^10,11^. It is clear that children whose mother drinks alcohol during pregnancy are prone to have a higher rate of epilepsy than does the general population, in which the prevalence of epilepsy is less than 1%^12^. These results from human studies strongly suggest that prenatal alcohol exposure can significantly increase the rate of epilepsy in children prenatally exposed to alcohol.

A number of animal studies have been conducted to determine the link between developmental ethanol exposure and the susceptibility to seizure. Results from several laboratories by using rodent models strongly suggested that ethanol exposure during early developmental stages predisposes the offspring to seizure^13–19^. However, conflicting results on whether developmental alcohol exposure predisposes the offspring to seizures were also reported by other laboratories using rodent models^20–24^. It is clear that while rodent seizure models have significantly contributed to our understanding of effects of developmental ethanol exposure on the seizure, the results from these studies are controversial. To further our understanding of the effects of fetal alcohol exposure on the vulnerability to epileptic seizures, elucidate the underlying mechanisms and develop novel treatments, additional animal models are needed. In addition, the comparison between various animal models is also critical for elucidating the mechanisms underlying neurologic dysfunctions induced by developmental alcohol exposure and for uncovering the evolutionarily conserved mechanisms of epileptogenesis. Zebrafish represent a powerful novel experimental model for this purpose.

Zebrafish (Danio rerio) are emerging as a promising animal model to study various brain dysfunctions, including epilepsy because it possesses several characteristics that are not offered by other traditional models. For example, the rapid development of zebrafish (days as opposed to weeks) makes it a great model for early developmental exposure. The tiny size of zebrafish larvae (~4 mm), and a large number of embryos (~250) produced from a single mating make it possible to test large numbers of animals at various experimental conditions simultaneously. Zebrafish larvae are also useful for pharmacological and toxicological screens, as they are permeable to small molecules^25,26^. In addition, a high degree of conservation of the nervous system is present between zebrafish and mammals, making comparative studies possible^27,28^. Furthermore, the use of larval zebrafish as a model organism in behavioral studies has been rapidly expanding due to the development and availability of advanced analytical platforms invented to measure locomotor activity in larval zebrafish^29–33^.

In this study, zebrafish was used as a model to investigate the effect of alcohol exposure during early developmental stage on the vulnerability to epileptic seizures induced by PTZ, a very well characterized chemical convulsant. To this end, zebrafish embryos at 3 hpf were exposed to ethanol at various concentrations for 21 hours. Control and ethanol-exposed seven-days-old zebrafish larvae were challenged with PTZ. The seizure behavior of larval fish was monitored and recorded using a digital camera and analyzed using EthoVision XT 11, a professional behavior tracking software. The results from this study demonstrated for the first time that ethanol exposure during early embryonic stage can decrease the threshold for chemically induced seizures and increase the severity of seizure behavior in larval fish.

## Results

### Embryonic ethanol exposure increased the percentage of larval zebrafish exhibiting a seizure behavioral response induced by PTZ

**Figure 1:**
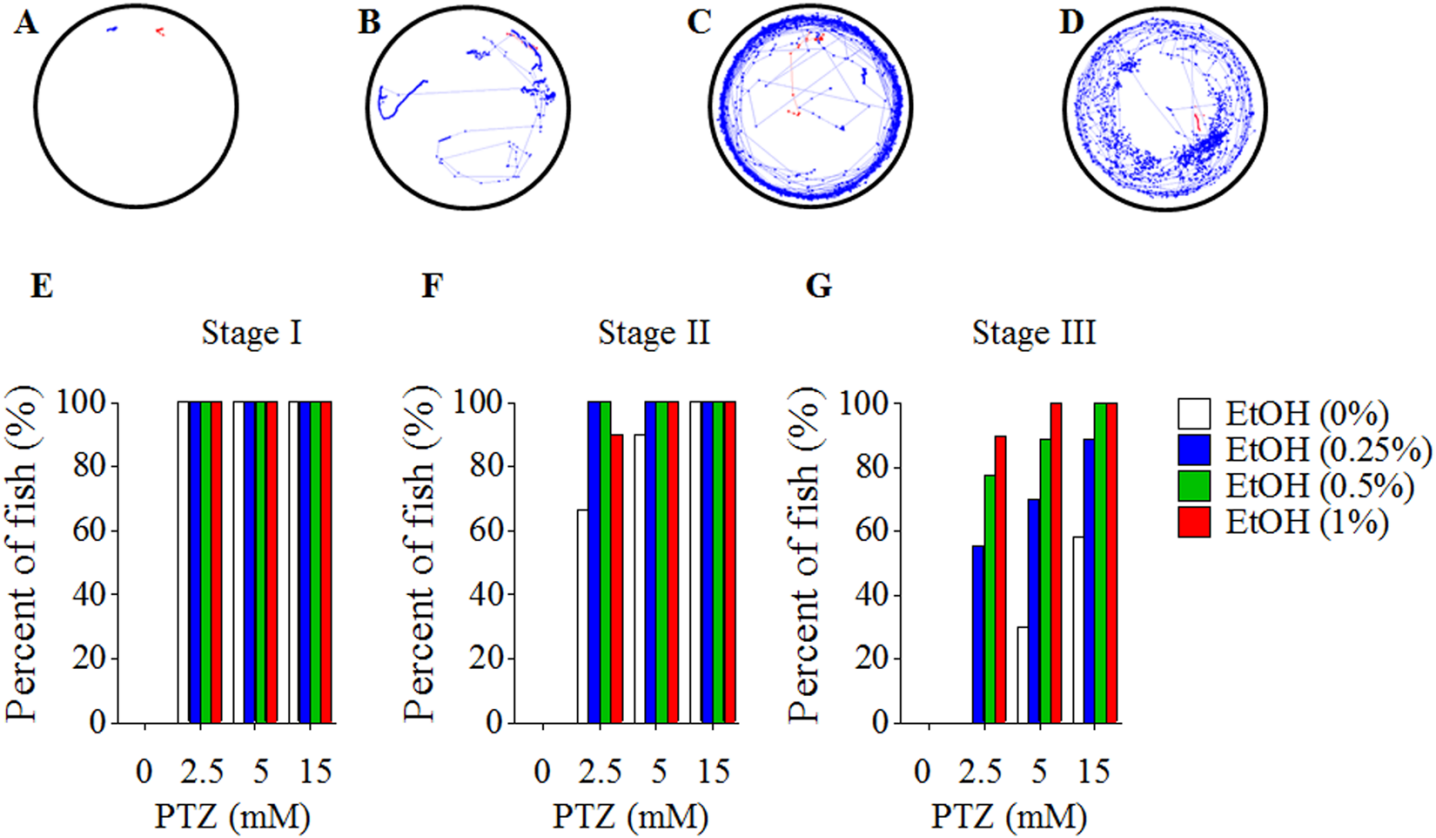
Percentage of fish with embryonic ethanol exposure exhibiting a seizure behavioral response induced by PTZ. (**A**–**D**) Representative trajectory plots of zebrafish larvae displaying three distinct seizure stages. (**A**) 0 mM PTZ, without discernable seizure behaviors; (**B**) 2.5 mM PTZ, typical stage I seizure behaviors; (**C**) 5 mM PTZ, typical stage II seizure behaviors; (**D**) 15 mM PTZ, typical stage III seizure behaviors. (**E**–**G**) Percentage of zebrafish with embryonic ethanol exposure reaching a defined seizure stage after exposure to PTZ for 15 min. (**E**) Stage I; (**F**) Stage II; (**G**) Stage III. At least 10 larvae were used for each treatment.

PTZ model is one of the most widely used animal seizure models for antiepileptic drug (AED) discovery and elucidation of the mechanisms underlying seizure^34–36^. Studies have indicated that PTZ can elicit seizures in zebrafish larvae, and a sequence of seizure-like behaviors has been well established and defined in 7 dpf zebrafish larvae by behavioral and electrographic studies, as well as by pharmacologic studies using valproate, diazepam and other antiepileptic drugs^34^. PTZ-induced seizures in 7 dpf zebrafish larvae, as well as in adult zebrafish have been demonstrated to be inhibited in a concentration-dependent manner by common antiepileptic drugs^37–39^. PTZ-induced seizure in zebrafish can generally be categorized into three stage patterns based on their advancement in locomotor behavior^34^. Using this well-established and validated 7 dpf zebrafish larvae PTZ-seizures model, we found that, at stage I, fish are characterized by a significantly general increase in locomotor activity (Fig. 1B). A stage II PTZ-induced seizure in zebrafish consists of an easily identifiable, rush, circular swimming motion, which is clearly distinguishable from the generalized increased activity observed at stage I (Fig. 1C). At stage III, zebrafish experiences a series of brief clonus-like convulsions and eventually progresses to a loss of position and remains immobile for several seconds (Fig. 1D). To determine whether embryonic exposure to ethanol increase the susceptibility to PTZ-induced seizure, zebrafish embryos were exposed to ethanol at concentrations ranging from 0.25 to 1% from 3 to 24 hpf, a period in zebrafish that encompasses both gastrulation (5.25 to 10 hpf) and neurulation (10 to 24 hpf) which falls within the first trimester of human gestation^40^. Control and ethanol-exposed 7 dpf zebrafish larvae, in which most of the main neuronal clusters and axon tracts have been formed^41^, were exposed to PTZ at the concentrations of 2.5, 5 or 15 mM. The seizure behavior of larval fish was monitored and recorded using a digital camera and analyzed using EthoVision XT11, a professional behavior tracking software. We found that seizure behavior was not observed in larvae that were not exposed to PTZ, regardless of ethanol-exposure at an embryonic stage. All larvae with or without prior embryonic ethanol exposure exhibited a stage I response when they were exposed to 2.5, 5 or 15 mM PTZ (Fig. 1E). Embryonic exposure to ethanol resulted in an increased seizure response at stage II and III in an ethanol concentration-dependent manner as compared with larvae without prior embryonic ethanol exposure and treated with the corresponding concentration of PTZ (Fig. 1E–G). When treatment with 2.5 mM PTZ, 65% larvae without prior ethanol exposure exhibited stage II seizure response but none of them exhibited stage III seizure. However, stage III seizure behavior was observed in 55%, 75% and 90% larvae with prior embryonic exposure to 0.25, 0.5 or 1% ethanol, respectively, indicating that the larvae with prior embryonic ethanol exposure can reach a stage III seizure when they were exposed to low concentration of PTZ (e.g., 2.5 mM), a concentration that cannot elicit stage III seizure at any larvae without prior ethanol exposure (Fig. 1G). When exposure to 5 mM PTZ, 90% and 28% larvae without prior embryonic ethanol exposure exhibited stage II and III seizure behavior, respectively. All larvae with prior embryonic ethanol exposure exhibited stage II seizure when exposed to 5 mM PTZ while 68%, 86% and 98% larvae with prior embryonic exposure to 0.25%, 0.5, and 1% ethanol, respectively, exhibited stage III seizure. It is noteworthy that embryonic exposure to 2.5% ethanol increased the percentage of larvae that exhibited stage III seizure to 70% in the presence of 5 mM PTZ, as compared to 30% in the larvae without prior ethanol exposure. When treatment with 15 mM PTZ, all larvae with or without prior ethanol exposure exhibited stage II seizure while 55% larvae without prior ethanol exposure, and 86% larvae with prior exposure to 0.25% ethanol exhibited stage III seizure. All larvae with prior exposure to either 0.5 or 1% ethanol exhibited stage III seizure behavior.

### Embryonic ethanol exposure increased the total distance traveled in larval zebrafish exposed to PTZ

**Figure 2:**
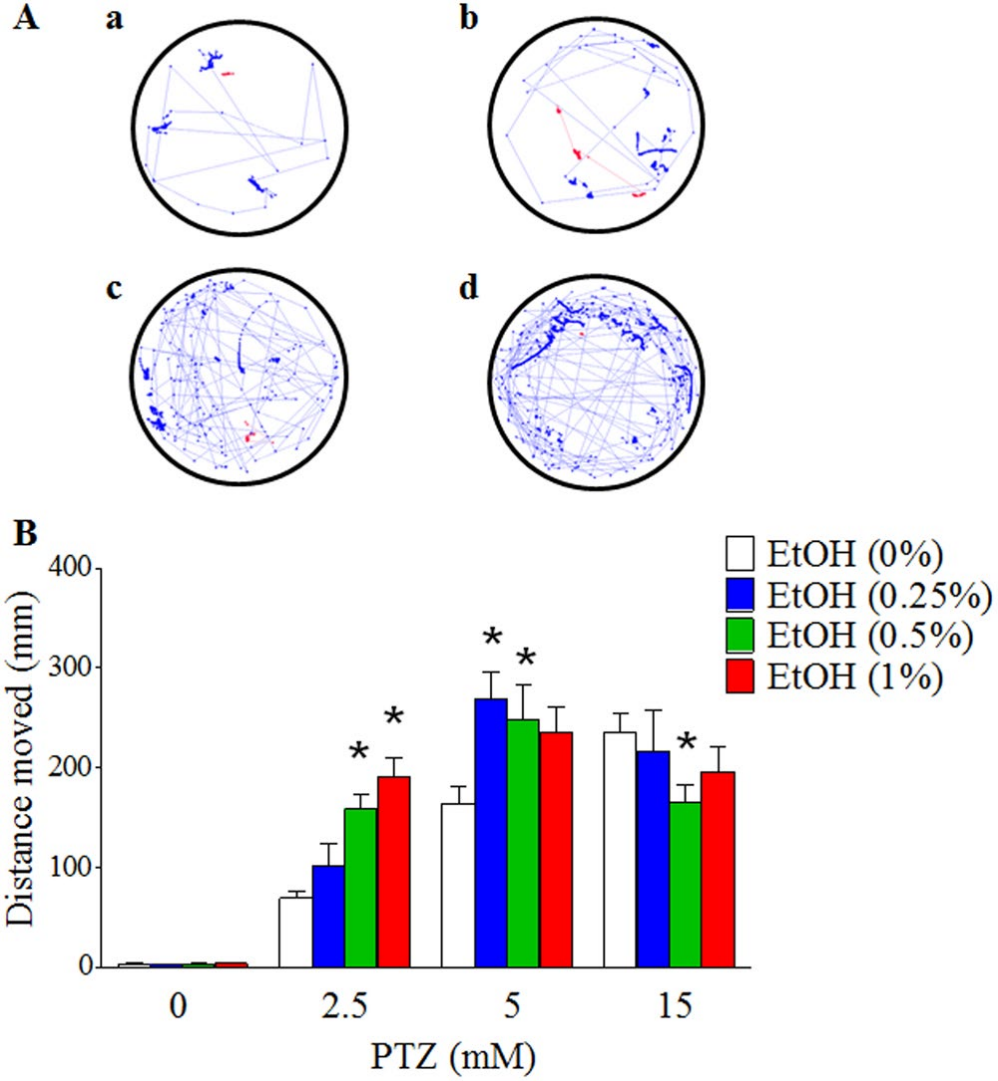
Effect of embryonic ethanol exposure on the total distance traveled in zebrafish exposed to PTZ. (**A**) Representative trajectory plots of zebrafish larvae exposed to different concentrations of ethanol and 2.5 mM PTZ. (a) Control; (b) 0.25% (v/v) EtOH; (c) 0.5% (v/v) EtOH; (d) 1% (v/v) EtOH. (**B**) Embryonic ethanol exposure increased the total distance traveled in zebrafish exposed to 2.5 or 5 mM PTZ. At least 10 larvae were used for each treatment. Data represent the mean ± SEM. *p < 0.05 vs. corresponding control.

As defined above, both stage I and II seizures exhibit increased movement while stage III seizures show a decreased movement. Analysis with EthoVision after 15 min treatment with PTZ showed a significant alteration in the distance traveled in the larvae with prior ethanol exposure (Fig. 2A,B). Larvae exposed to 2.5 mM PTZ showed a significant increase in distance moved in an ethanol concentration-dependent manner. The larvae with prior exposure to 0.5% ethanol traveled at least twice the distance as the larvae without prior ethanol exposure (Fig. 2B). At a concentration of 5 mM PTZ, all larvae with prior ethanol exposure showed a general increase in distance traveled. However, the distance traveled in the group with prior exposure to a lower concentration of ethanol traveled more distance than that exposed to higher concentration of ethanol, consistent with the fact that more larvae with prior exposure to higher concentration of ethanol reach stage III seizure. In the presence of 15 mM PTZ, all ethanol-exposed groups showed a general decrease in distance moved (Fig. 2).

### Embryonic ethanol exposure increased the duration of time spent in a general active state or highly mobile state in larval zebrafish exposed to PTZ

**Figure 3:**
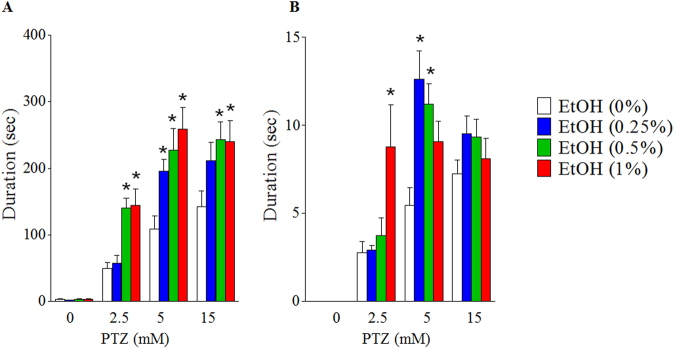
Effect of embryonic ethanol exposure on the duration of time spent in a general active state or highly mobile state after exposure to PTZ. (**A**) Duration of time spent in a mobile state after exposure to PTZ. (**B**) Duration of time spent in a highly mobile state after exposure to PTZ. At least 10 larvae were used for each treatment. Data represent the mean ± SEM. *p < 0.05 vs. corresponding control.

Although stage III seizure is characterized by convulsive behavior without distance movement, it exhibits stationary body movement. In addition to detecting distance moved, the latest version of EthoVision software, EthoVision XT 11, can also analyze other mobility parameters, including stationary body movement. In this study, the mobility duration, a parameter incorporates both stage II and Stage III body movement, was analyzed in the larvae exposed to PTZ. As shown in Fig. 3, at 5 mM PTZ, larvae with prior exposure to 0.25% ethanol showed a significant increase in mobility duration (Fig. 3A) as well as the duration of time spent in a strongly mobile state, which was mostly observed at stage II seizure (Fig. 3B). Larvae that were exposed to 0.5% ethanol at embryonic stage showed dramatic increases in mobility when they were exposed to all PTZ doses tested (Fig. 3A). The fish in this group also spent significantly more time in a strong mobile state at 5 mM PTZ (Fig. 3B). The larvae with prior embryonic exposure to 1% ethanol showed a significant increase in mobility duration at 2.5, 5 or 15 mM PTZ and the duration of strong mobility at 2.5 mM PTZ but not at 5 or 15 mM PTZ (Fig. 3B), indicating that these larvae were undergoing Stage III convulsive motions at higher PTZ doses. Similarly, members of two other dose groups of ethanol (0.25% and 0.5%, v/v) that have shown a significant increase in strong mobility duration at 5 mM PTZ didn’t show a significant increase in strong mobility at 15 mM PTZ doses (Fig. 3B), thereby indicating that these larvae may also undertake convulsive motions at highest PTZ dose. Although distance moved not significantly increased compared with control treatment, the members of the highest ethanol group (1%, v/v) showed a significant increase in mobility at higher PTZ doses, but its increase in duration of strong mobility is generally reduced, confirming that these larvae were undergoing relative convulsive motions.

### Embryonic ethanol exposure resulted in a significant reduction in seizure latency in larval zebrafish exposed to PTZ

**Figure 4:**
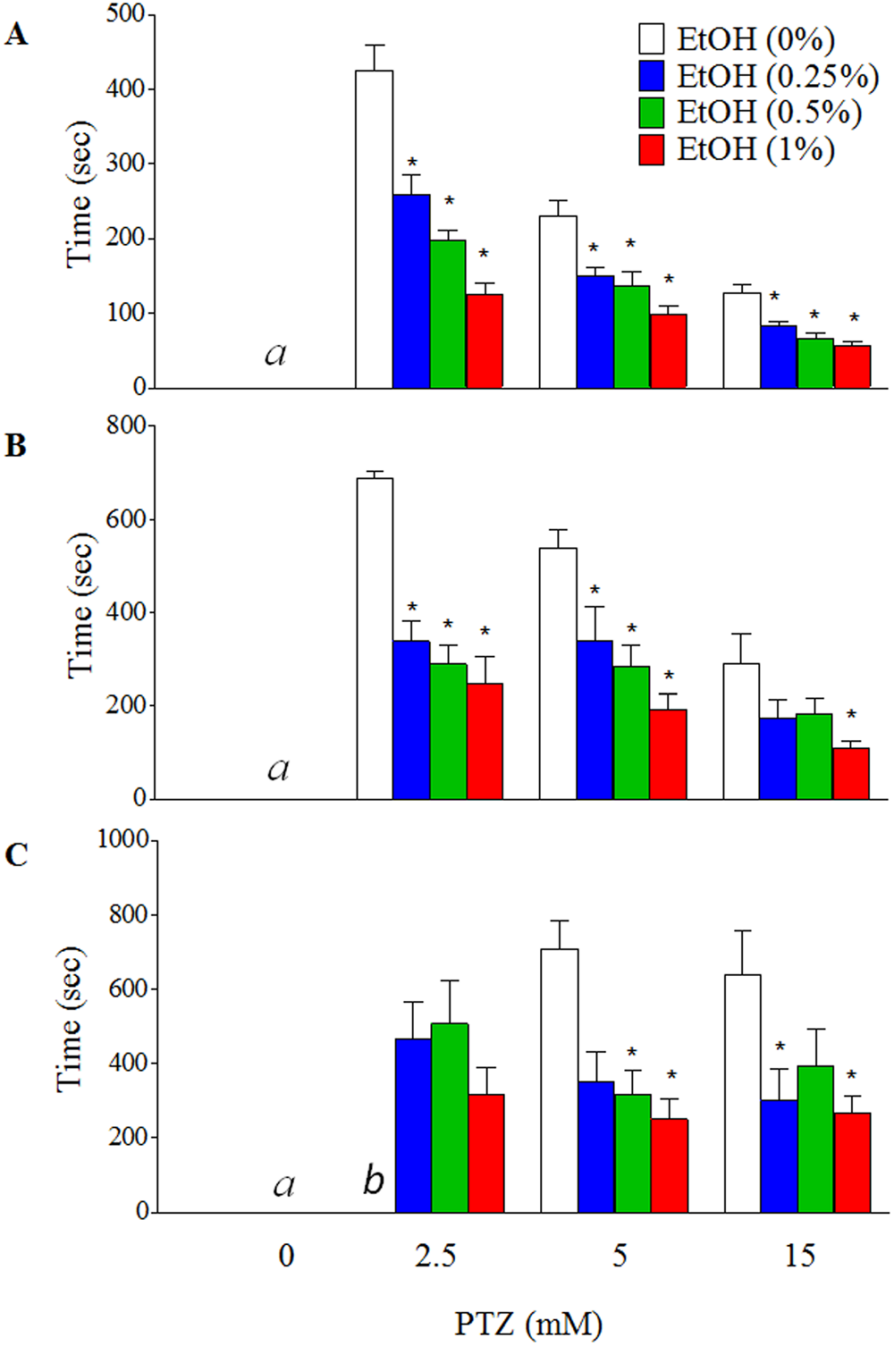
Effect of embryonic ethanol exposure on the latency to the first sign of PTZ-induced seizure behaviors for three different stages. (**A**) Stage I. (**B**) Stage II. (**C**) Stage III. ^*a*^No fish had a seizure response in the absence of PTZ. ^*b*^No fish in this group was able to reach stage III seizure. At least 10 larvae were used for each treatment. Data represent the mean ± SEM. *p < 0.05 vs. corresponding control.

The time required for the onset of the first sign of Stage I, II and III seizures in the larvae after PTZ challenge was used to determine the seizure latency. We found that, in control fish that were not exposed to ethanol embryonically, the seizure latency in stage I, II were 425 sec and 680 sec, respectively, after they were exposed to 2.5 mM PTZ, and were 140 sec and 300 sec, respectively, after they were exposed to 15 mM PTZ (Fig. 4A and B). In control fish that were not exposed to ethanol, the seizure latency in stage III was 700 sec for 5 mM PTZ and 640 sec for 15 mM PTZ (Fig. 4C). All larvae exposed to ethanol embryonically showed a significant time reduction in stage I, II or III seizure latency after exposed to PTZ. At high PTZ concentration (15 mM), a stage I, II or III seizure was observed within 60 sec, 100 sec, and 250 sec, respectively, in the group exposed to 1% ethanol embryonically (Fig. 4). The embryonic exposure to 1% ethanol also resulted in a stage II seizure behavioral response within 100 sec following the exposure to 5 mM PTZ (Fig. 4). Significant time reductions of seizure latencies were also observed across other dose combinations of ethanol and PTZ. After exposure to 2.5, 5, or 15 mM PTZ, a significant time reduction in stage I, II or III seizure latency (*p* < 0.05) were observed in the larvae that were embryonically exposed to the lowest concentration of ethanol (0.25%) (Fig. 4). While no stage III seizure behavior was observed in control larvae that were exposed to 2.5 mM PTZ, exposure to this low dose of PTZ resulted in a stage III seizure in larvae that were embryonically exposed to ethanol (Fig. 4).

## Discussion

In this study, we demonstrate that embryonic exposure to ethanol at concentrations as low as 0.25–1.0% can increase the vulnerability of larval zebrafish to the PTZ-induced seizures. We found that embryonic ethanol exposure increased the percentage of larval zebrafish exhibiting typical stage II and III seizures induced by PTZ. Ethanol exposure during the early developmental stage also significantly increased the total distance traveled in larval zebrafish exposed to PTZ. In addition, embryonic ethanol exposure increased the duration of time spent in a general active state or highly mobile state in larval zebrafish exposed to PTZ. Ethanol exposure also resulted in a significant reduction in seizure latency in larval zebrafish exposed to PTZ. These results demonstrate that ethanol exposure during early embryonic stage can reduce the threshold for chemically induced seizures and increase the severity of seizure behaviors in larval fish.

Previous studies using rodent models have contributed significantly to our understanding of the effects of developmental ethanol exposure on the susceptibility to seizure. Yanai and colleagues found that the threshold for audiogenic seizures was decreased in the mice whose mothers drank alcohol during pregnancy^13,14^. Studies have also shown that alcohol exposure during the brain growth spurt facilitated PTZ-induced seizures, in the form of increased behavioral activity and electrographic epileptic activity^16^. It has also been reported that fetal alcohol exposure induced long-lasting increases in postnatal anxiety, tolerance to acute sedative/anesthetic effects of ethanol, and increases seizure susceptibility^15^. These studies are consistent with the results from our current studies and support the premise that exposure to ethanol during critical developmental stage predisposes the offspring to seizures later in life.

It is well known that interactions of environmental risk factors with genetic factors play an important role in the etiology of seizures^42–44^. Exposure to environmental risk factors early in life can result in profound changes in brain structure, which constitute the pathological basis of adult neurological diseases, including seizures. Early developmental exposure to nicotine^45^, lead^46^, methylazoxymethanol acetate^47^, hypoxia^48^, lamotrigine^49^, gamma rays^50^, methamphetamine^51^, methylmercury^52^, polychlorinated biphenyls^53^ or domoic acid^29^ has been shown to initiate or promote the development of seizures. In addition, improved maternal care has been shown to reduce seizure susceptibility in rodents^54–56^.

The mechanism by which early alcohol exposure increases the susceptibility to PTZ-induced seizures later in life is not well understood. However, it is well known that ethanol exposure during the early developmental stages impaired brain development and functions, including increased neuronal cell death, impaired neuronal migration, altered neurotransmitters and their receptors, and disrupted neuronal circuitry, which could predispose the developing offsprings to PTZ-induced seizures^16,18,57,58^. It was reported that altered hippocampal structure is one of the prominent characteristics in the offspring of an alcoholic mother^59,60^. Studies in animals revealed that developmental ethanol exposure produces long-term hippocampal cell loss^61,62^. It was well-established that hippocampus plays a critical role in seizure occurrences^63,64^. A direct experimental observation from Bonthius and co-works suggested that the permanent disruption of the hippocampal formation by ethanol through selected cell loss contributes heavily to the reduced seizure threshold^65^.

Alcohol-induced impairments in neurotransmitters and their receptors may also play a role in ethanol-induced increased vulnerability to epileptic seizures^57,66^. One of the neurotransmitter receptors that is targeted by ethanol is γ-aminobutyric acid (GABA) receptor^67–70^. It has been demonstrated that both chronic and acute ethanol administration resulted in persistent alterations in GABA receptor subunit composition, localization, and function^67–72^. These changes in the physiology of GABA receptors occur not only in adult animals but also in developing animals exposed to ethanol during the brain development^73,74^. It is well documented that animals with genetic defects of the GABA receptor are predisposed to seizures^75,76^. Considering the fact that PTZ is a competitive antagonist of the GABA receptor^77^ and that alcohol can disrupt GABA receptor, it is likely that alcohol-induced alteration of GABA receptor contributes to the reduced seizure threshold observed in this study. It is also noteworthy that the GABA systems have been shown to develop early during embryogenesis and are present in larval zebrafish^78^, providing a physiological basis for the possible involvement of GABA receptor in ethanol-induced vulnerability to epileptic seizures in zebrafish larvae. However, the role of GABA receptor in the ethanol-induced reduction of seizure threshold in zebrafish larvae would need to be explored further.

While multiple studies from other laboratories using rodent models and our study using zebrafish strongly suggested that ethanol exposure during the development increases the vulnerability to epileptic seizures, there were other studies that have found that developmental exposure to ethanol does not make the rats more prone to seizures later in life^20–24,79^. One of the proposed explanations for these inconsistent results is the timing of the alcohol exposure^16^. While those studies in rodent model that have found that developmental exposure to ethanol does not predispose the offspring to seizures later in life administered alcohol during the prenatal period^20–24,79^, other studies using rodent model that have demonstrated that early ethanol exposure increased the vulnerability to seizures administered alcohol postnatally^15,16,65^. It is well known that the consequences of alcohol exposure during development depend heavily on the timing of the exposure. Administration of a similar dose of alcohol at different developmental stages can have substantially different physiologic, pathologic, and behavioral effects^80–83^. However, our present study demonstrated that exposure of the zebrafish embryos to ethanol at 3–24 hpf, which falls within the first trimester of human gestation and prenatal stage of mouse and rat development, can increase the vulnerability to seizures in larvae, indicating that the factors other than the timing of the alcohol exposure might also contribute to the inconsistent results.

To the best of our knowledge, the rodent was the only animal model employed in all published studies examining the effect of alcohol exposure during development on the vulnerability to epileptic seizures later in life. Controversial results from previous studies using rodent models warrant further investigation using additional animal models. For this purpose, zebrafish represents a promising novel model for studying seizure behavior and epilepsy in the offspring of early ethanol exposure. That the seizure behaviors can be further analyzed using a high-speed locomotion tracking system allows rapid quantification of behavior with minimal human effort. Indeed, zebrafish has been successfully used as a behavioral model to study the effects of alcohol on locomotor activity, and these studies showed that ethanol administration produced a biphasic dose-response pattern, where lower doses increased activity, and higher doses decreased activity, in both larval^84–87^ or adult fish^88,89^. Studies have also indicated that PTZ can elicit seizures in zebrafish larvae, and a sequence of seizure-like behaviors has been well established and defined in zebrafish^34^. Comparing our findings in zebrafish with previously published data in rodent models may enable a better understanding of the effects of developmental ethanol exposure on the susceptibility to seizures.

In conclusion, our present study indicated that early embryonic exposure increased the percentage of larval zebrafish exhibiting typical stage II and III seizure and resulted in a significant reduction in stage I, II and III seizure latency in an ethanol concentration-dependent manner. Embryonic exposure to ethanol also significantly increased the severity of PTZ-induced seizures in larval zebrafish, as demonstrated by increased total distance traveled and the duration of mobility. This is the first demonstration that ethanol exposure during early embryonic stage can reduce the threshold to chemically induced seizures and increase the severity of seizure behavior in larval fish. These findings suggest that, in addition to causing FAS, early embryonic exposure to ethanol may enhance the biological risk for chronic diseases later in life. Future studies to analyze the alteration in the expression of genes that predispose the offspring to seizures and the potential epigenetic regulation of these genes in zebrafish embryos and larvae exposed to ethanol during early development will further elucidate the molecular mechanism underlying the embryonic ethanol exposure-induced vulnerability to seizures.

## Methods

### Zebrafish

Zebrafish (*Danio rerio*) of wild-type AB strain were obtained from Zebrafish International Resource Center (ZIRC; Eugene, OR) and maintained in a zebrafish housing system (Aquaneering; San Diego, CA). Fish were fed twice a day with commercial flake food (Tetra, Daleville, VA) and with live brine shrimp to incite optimal egg production. Females and males in a ratio of 1:2 were transferred into crossing tanks in the evening before spawning induction. Newly fertilized eggs were raised at 28 °C in embryo water (Milli-Q water with 60 mg/L Instant Ocean). All protocols used in this study were approved by the University of Louisville Institutional Animal Care and Use Committee, and all experiments were conducted in accordance with the relevant guidelines and regulations.

### Ethanol treatment of embryos

Embryos were exposed to ethanol at concentrations ranging from 0.25–1% (v/v) during 3–24 hours post fertilization (hpf). Stable ethanol levels were maintained by using a screw cap for each vial. After ethanol exposure, the embryos were rinsed three times and raised to 7 days of post fertilization (dpf) in a 28 °C incubator before they were exposed to PTZ.

### PTZ challenges

7-day-old larvae from control and treatment groups were transferred to individual wells of 96-well plates (VWR scientific) with 50 μL fresh Ringers solution. Any fish with growth retardation or morphology abnormalities was excluded from the study. The plate was placed on the stage of an Olympus (SZH) stereo microscope system equipped with an RGB camera which was connected to a computer running EthoVision XT 11 behavioral tracking software (Noldus Information Technology Inc., Leesburg, VA). Before they were exposed to PTZ, a 3 min baseline trial was tracked and recorded in larvae at control medium. The medium was then replaced with one of three concentrations of PTZ (2.5, 5, 15 mM). The response was recorded for a total of 18 min with an additional 3 min trial after the 15 min of exposure had lapsed.

### Data analysis

Recordings of the larvae after the PTZ exposure were analyzed for behavioral changes and seizures. The distance moved and the mobility parameters were analyzed by using EthoVision XT 11 tracking program, and the baseline parameters were subtracted out before calculation. Time to reach the first definitive stage I, II or III seizure (seizure latency) was also determined. A minimum of 10 individuals per ethanol/PTZ combination was used in all calculations. Two-way analysis of variance (ANOVA) followed by Bonferroni means comparison test (Prism version 5; GraphPad Software Inc. San Diego, CA) were used to analyze the compounding effects of both ethanol and PTZ on the larval fish.
